# A Review of Deep Learning Methods for Antibodies

**DOI:** 10.3390/antib9020012

**Published:** 2020-04-28

**Authors:** Jordan Graves, Jacob Byerly, Eduardo Priego, Naren Makkapati, S. Vince Parish, Brenda Medellin, Monica Berrondo

**Affiliations:** Macromoltek, Inc, 2500 W William Cannon Dr, Suite 204, Austin, Austin, TX 78745, USA

**Keywords:** antibody, antigen, machine learning, deep learning, neural networks, binding prediction, protein–protein interaction, epitope mapping, drug discovery, drug design

## Abstract

Driven by its successes across domains such as computer vision and natural language processing, deep learning has recently entered the field of biology by aiding in cellular image classification, finding genomic connections, and advancing drug discovery. In drug discovery and protein engineering, a major goal is to design a molecule that will perform a useful function as a therapeutic drug. Typically, the focus has been on small molecules, but new approaches have been developed to apply these same principles of deep learning to biologics, such as antibodies. Here we give a brief background of deep learning as it applies to antibody drug development, and an in-depth explanation of several deep learning algorithms that have been proposed to solve aspects of both protein design in general, and antibody design in particular.

## 1. Introduction

In this paper, we outline the deep learning techniques that are starting to be applied to the field of antibody design and their results. We outline current challenges in three areas of antibody design: (1) modeling of structure from sequence, (2) prediction of protein interactions, and (3) identification of likely binding sites. We then touch on the deep learning techniques and algorithms that have been developed towards antibody design in each of these areas. As these three challenge areas have analogues in the general protein space, we additionally describe deep learning approaches for similar problems among proteins more broadly, with the understanding that these approaches may be applicable to the narrower domain of antibody engineering. We also describe the dataset and benchmarks available that could aid in the development and comparison of methods within this field. We conclude with a comparison of these methods and touch on future directions.

Monoclonal antibody therapeutics have become an increasingly popular approach for drug development against targets and indications where small-molecule-based approaches have proven insufficient. With this increase in focus has come the creation of a number of new methods for improving and refining the antibody development pipeline. Innovations in constructing display libraries (phage and yeast) have accelerated the candidate discovery timeline and reduced the challenges associated with downstream development of therapeutic leads. A significant goal of current lead development research has been to reduce the necessity of downstream lead optimization steps such as improvements to the solubility and immunogenicity of the candidates, along with the mitigation of other developability concerns. Other research has expanded the mechanisms of action of potential antibody therapeutics by adding additional functional domains to create bispecific and Fc effector antibodies, and by exploring different constructs for their unique properties, such as single-chain variable fragments and camelid-derived nanobodies.

While these in vitro innovations have shortened timelines and have improved different steps throughout of the development pipeline, there have been a new class of innovations surrounding the *in silico* engineering and design of antibody candidates. These approaches attempt to harness advances in computational processing power to reduce the cost and increase the speed of lead candidate generation. The advantages of an *in silico* pipeline would include rapid and cheap scaling of candidate generation, the ability to develop antibodies against challenging antigens, and the application of rational design principles. In contrast to traditional methods of candidate generation such as hybridoma or phage display, an *in silico* pipeline promises cheaper and faster drug development. However, conventional *in silico* methods have yet to fully deliver on these promises. Here, we present deep-learning-based approaches that appear to demonstrate greater success than conventional methods with respect to the key challenges of computational antibody design.

## 2. Antibodies

**Figure 1:**
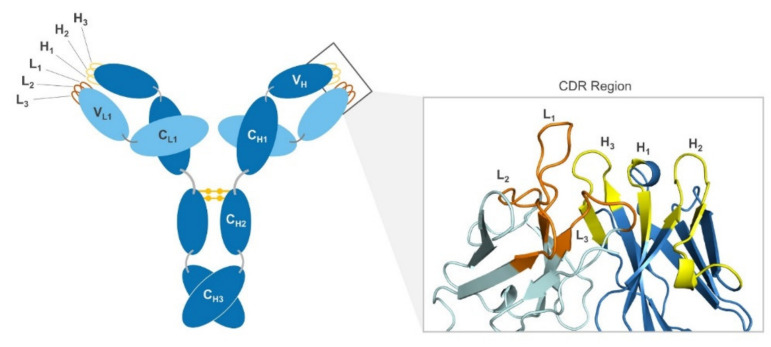
Schematic of antibody and ribbon diagram of variable region. The heavy chain (H) of the antibody is depicted in dark blue, while the light chain (L) is shown in light blue. Both chains show labels C for constant region and V for variable region. The complementarity-determining region (CDR) is shown as orange loops on the light chain and yellow loops on the heavy chain. On the right, a ribbon diagram of a CDR is shown with light and heavy chain CDR loops highlighted and labeled (PDB: 1A4J).

Antibodies are a type of protein produced as an immune response to invading pathogens. They consist of four chains—two heavy chains and two light chains. The heavy chains include three constant domains and a variable domain, while the light chains have just one constant domain and one variable domain. The variable domains contain the antibody’s binding surface, or “paratope”. The paratope primarily consists of six distinct variable loops—three on the light chain (loops L1, L2, and L3), and three on the heavy chain (loops H1, H2, and H3) (Figure 1). This region, also called the complementarity-determining region, or CDR, is what allows an antibody to bind a target with high specificity [1]. The area is large enough to accommodate many unique contacts, which is part of what allows for such high specificity—especially as compared to typical small molecules, which are able to accommodate far fewer contacts and thus tend to have a greater number of side-effect-causing off-target interactions. The substantial degree of variation between the CDR loops is significant, as the diversity of antibodies is part of what makes them effective binders for such a wide range of targets [1].

The specificity and broad applicability of antibodies make them the subject of much attention in medical research, and this has in turn attracted much attention to the study of antibodies computationally, or in silico.

In order to computationally analyze an antibody or predict its effectiveness, it is often necessary to generate a three-dimensional model. As traditional structure-determination methods, such as X-ray crystallography, Nuclear Magnetic Resonance (NMR), and Cryogenic Electron Mycroscopy (CryoEM), are laborious, time-consuming, and expensive, computational methods have emerged to generate structure predictions using chemistry and existing protein fold data. Several groups have been able to accurately predict antibody structures for a set of benchmarks, but the modeling of the H3 CDR loop continues to present a significant challenge [2]. The biological process which generates the H3 loop is unique relative to the other CDR loops. The bulk of the loop is encoded in its own gene, separate from the genes which code for the rest of the antibody sequence. Whereas other CDR loops exhibit much less variation and can even be reasonably separated into canonical structural clusters, the H3-encoding gene is actively mutated in isolation before being recombined with the rest of the gene sequence in a process called V(D)J recombination, which creates both sequential and conformational hypervariability among these loops [3,4]. The extreme variety of loop sequences introduced by this process makes homology to similar loops nearly impossible; there is rarely sufficient homology data to predict structure. This presents a substantial challenge which makes evident the need for new approaches.

**Figure 2:**
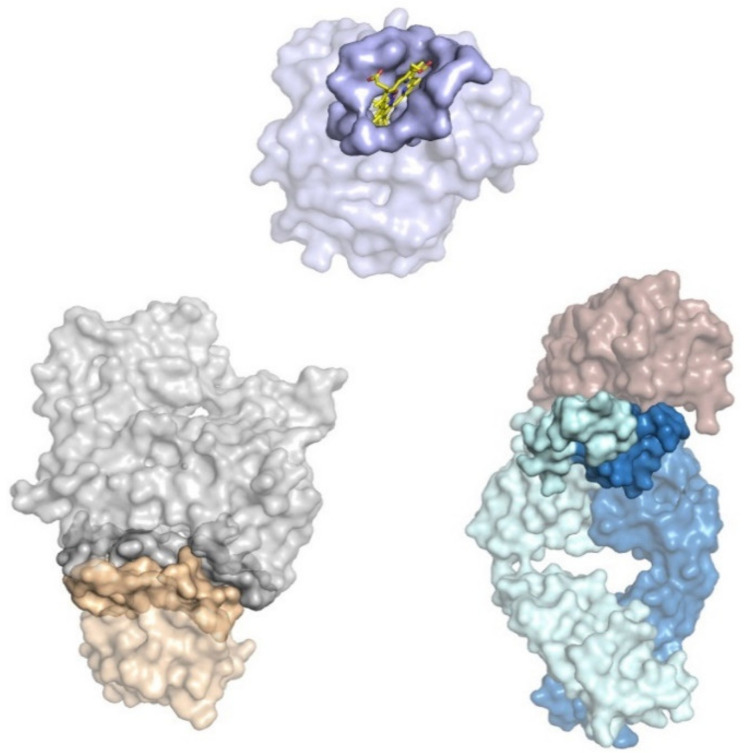
Protein interfaces surface representation. Binding interfaces represented in solid surfaces. At the top, myoglobin (PDB: 1MBN) is shown in purple, with a heme molecule (yellow sticks) bound and the binding pocket in a solid surface. At the bottom left, beta-actin (light gray, top molecule) is shown bound to profilin (wheat, bottom molecule) with their protein–protein interface (PPI) in solid surface (PDB: 2BTF). On the bottom right, a TSH receptor (brick red, top molecule) and antibody (light blue for light chain, dark blue heavy chain, bottom molecule) complex is shown. Only the antibody CDR is highlighted as a solid surface (PDB: 2XWT).

Another challenge in computational antibody development is interface prediction. Typically, the interface between two proteins consists of several well-conserved residues that form a tight interaction. The reason for this is that typically any two interacting proteins will have co-evolved over the span of many generations. An antibody’s interaction with its antigen does not benefit from such a long shared history. Antibodies are ad-hoc binders generated “on the fly” to address a foreign pathogen. Although antibody-antigen interactions do fall under the general umbrella of protein–protein interactions (PPI), it has become increasingly apparent that antibody–antigen interactions and their interfaces are distinct, with unique properties that reduce the applicability of general protein interaction prediction to the antibody space. (Figure 2) As only the antibody side of the interface undergoes its own, separate evolution, the antigen surface lacks many of the features associated with PPIs, including a lack of enrichment with non-polar and aromatic residues. These interfaces have fewer hydrophobic interactions, and are typically constructed of paratope aromatic hotspots surrounded by polar contacts created by short-chain hydrophilic residues [5]. Most of the cross-interface hydrogen bonds are created by sidechain–sidechain interactions and there are fewer backbone–backbone hydrogen bonds than in obligate PPIs and enzyme-inhibitor complexes [6].

## 3. What Is Deep Learning?

**Figure 3:**
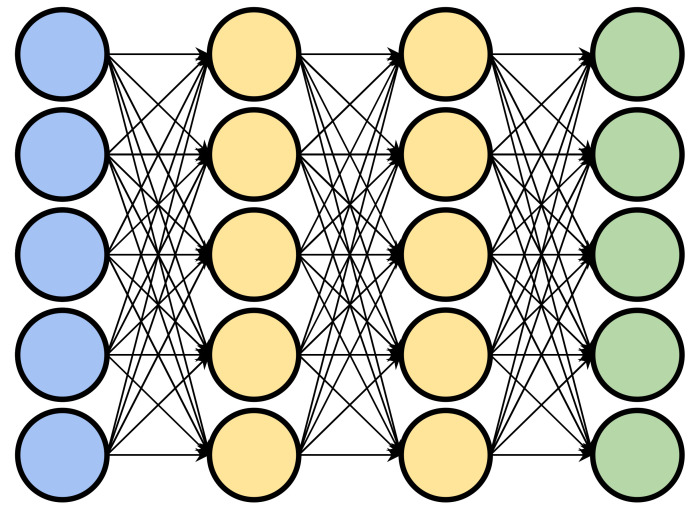
A representation of a deep learning neural network. Input layer in blue, output layer in green, and intermediate layers in yellow.

Deep learning is a subset of machine learning, concerned with algorithms that are particularly capable of extracting high level features from raw, low level representations of data. An example of this is an algorithm which extracts object curvature or depth from raw pixels within an image. Deep learning algorithms generally consist of artificial neural networks (ANN) with one or more intermediate layers. The intermediate layers of an ANN make the network “deep” and can be considered responsible for transforming the low-level data into a more abstract high-level representation. Each layer of the network consists of an arrangement of nodes, or “neurons”, which each take as input a set of weighted values and transform them into a single output value (usually by summing the weighted inputs). The resulting values are then passed on to nodes in subsequent layers. The input values for the first layer are chosen by the practitioner or model architect. In a biochemical context, these features can be hand-crafted values such as a protein’s volume, or lower-level values such as an amino acid sequence. In a “deep” network, the outputs of the first layer are passed through one or more intermediate layers before the final output layer which produces the final result. The intermediate layers allow the network to learn non-linear relationships between inputs and outputs by extracting successively higher level features and passing them along to subsequent layers (Figure 3).

In order for a neural network to transform an input into a desired output, it must be “trained”. Training a neural network happens by modification of the weights of the connections between nodes. In a “fully connected” network each node is connected to each node in subsequent layers. The output values from the nodes of the preceding layer are passed along weighted connections to the nodes of the subsequent layer. These weights of the connections are typically initially randomized, and as the network is trained, corrections are made by iteratively modifying the weights in such a way that the network is more inclined to produce the desired output from the given input. The correctness of a model is determined by a “cost function”, which provides a numerical measure of the amount of error in a model’s output. The choice of the cost function largely depends on the task of the network and functions as a proxy for minimizing or maximizing some metric which cannot be directly used for optimization since it is non-differentiable, such as classification accuracy. To determine the direction each weight must be changed in order to come closer to a desired output, the partial derivative of the cost function is computed with respect to the network’s weights [7]. Examples of cost functions include binary cross entropy used for binary classification tasks, or mean squared error, often used for regression tasks. By repeating this training protocol with many passes over the entire dataset, the model can be trained to identify and weigh features in the data that are broadly predictive of the end result. Like other machine learning methods, the use of a training, validation, and testing dataset is used to assess model performance. The training set is a subset of the data used to reinforce the model to the desired output, while the validation subset is used to prevent the network from overfitting by terminating training based upon some criteria computed from the validation set. A common criterion is early stopping, which terminates training once the performance on the validation set begins to diminish. The test subset, often termed the hold-out set, is used to analyze a trained model’s generalization capabilities by evaluating the model on unseen data samples.

Within the past decade, deep learning algorithms have shown super-human capabilities in competitive tasks such as Go and Chess [8]. While these methods have benefitted from a virtually infinite number of training examples, other methods have also seen human-level capabilities using human annotated datasets. Such applications include image classification and speech recognition where hand-crafted features have been replaced with features extracted within the internal layers of deep learning models. While the application domain between these methods is very different from biological, similarities exist between the data representation within these applications and those of biological data. Biological data is arguably more complex and the capability of these methods to learn rich, high level features makes them attractive methods for learning patterns within more complex data

### 3.1. Modeling (Sequence to Structure)

Protein crystal structures have been instrumental in current research surrounding protein–protein interactions, protein function, and drug development. While the number of experimentally determined protein crystal structures has grown significantly, it is dwarfed by the amount of sequence data that has been made available [9]. In the last several decades, the number of known protein sequences has climbed exponentially with the continued development of sequencing technologies. Due to this disparity, several three-dimensional structure modeling approaches have been created to bridge the gap between the availability of sequences and the shortage of known structures.

Current methods of protein structure modeling include homology modeling and ab initio modeling. In homology modeling, the sequence of the protein is compared to those of proteins with known structures. Closely related proteins or protein domains are used as structural templates for the corresponding region in the target sequence [8]. Ab initio modeling is used in situations where there are not similar sequences for which structures are known or are otherwise unsuitable for homology modeling. In this case, ab initio algorithms attempt to generate the three-dimensional structure of the protein using only its sequence. Typically, this is done by sampling known residue conformations and/or searching for known protein fragments (local protein structure) to use as part of the structure [10,11]. This is aided by tools like knowledge-based and empirical energy functions to select viable structures.

### 3.2. Interaction Prediction/Affinity Maturation/Docking

Antibody therapeutics are designed against a target protein. Therefore, it is critical to be able to understand and infer the binding behavior between the antibody and the target, such as whether or not the two proteins will have an energetically favorable interaction (interaction prediction), which residues will form the interaction interface and in what conformation (docking), or how certain amino acid substitutions will change the binding energy (affinity maturation). Docking algorithms attempt to solve the exact three-dimensional conformational pose between two or more interacting structures. Software to predict bound complexes of drug candidates has existed as far back as 1982 [12]. Originally used for small-molecule ligands where current standards include GOLD, DOCK, and AutoDock Vina, docking algorithms have expanded into the protein–protein domain with current standards including ZDOCK, ClusPro, Haddock, RosettaDock and several others [13,14,15,16]. Common to these methods are sampling techniques such as Monte Carlo or fast-Fourier transform, which aim to generate structural conformations that can be scored with a function which estimates the energetic favorability of two docked structures [17,18]. Interaction prediction algorithms which classify bound structures based upon energetic favorability can be used to filter candidates and narrow down the search space.

Another set of algorithms, named affinity maturation after the similar process in B-cell response, attempts to determine if mutations or modifications to the binding partners have an impact on binding affinity or energetic favorability or generate mutations or sequences which increase the binding affinity of the partners.

### 3.3. Target Identification (Epitope Mapping)

Target identification includes methods used to locate binding sites on proteins in the absence of knowledge about the protein’s binding partner. Since proteins exhibit specificity towards binding partners, this task is considerably difficult.

Antibody binding sites (epitopes) can be classified into two categories T-cell epitopes and B-cell epitopes. B-cell epitopes can further be divided into linear and discontinuous [19]. While T-cell epitope prediction methods have seen greater success, B-cell epitope prediction remains a difficult and unsolved problem [20]. Despite being theorized as an unsolvable problem, several methods have been proposed and claim moderate success [21].

## 4. Why Deep Learning for These Problems?

Traditional approaches to these problems have tended to rely on theoretical energy functions (physics-based) or statistical (knowledge-based) energies. However, no force field fully captures the complex interactions present with biological molecules and physics-based simulations require immense computational time. Deep learning offers the ability to perform efficient high-level feature-extraction of properties which are not otherwise captured with current energy functions.

In recent years, solutions to computer vision problems have seen significant increases in success due to geometric deep learning and convolutional neural networks [22,23]. Convolutional neural networks utilize sliding filters across the input domain in the form of a weight matrix which transforms the input to degrees of filter matching for each filter. This sliding filter is analogous to a normal network layer which only receives input from a subset of the neurons in the previous layer. The recent advancement seen in computer vision using these techniques is particularly significant for structure-based deep learning methods, as such problems can be framed as computer vision problems. By choosing to represent a protein structure as, for example, a graph, manifold or 3D voxel grid, it can be rendered compatible with these convolutional filters. For graph-based approaches, atoms or residues may be represented as nodes, with interactions between them taking the form of edges. Voxel-based methods discretize the protein into a grid, with grid points annotated with compositional content, such as atomic makeup [24]. Manifold methods may be used for representing the surface of a protein as relative degrees of curvature and energetic properties across the domain.

Additionally, recurrent neural networks—often used in language processing tasks—can be utilized by framing sequence data as a language with a unique vocabulary [25]. Recurrent neural networks typically use an array-like structure of layers where layer inputs are either direct input values, such as the letters in a word, or hidden inputs, which are outputs from a previous layer in the array. The hidden nodes allow context to propagate along the array and subsequent input values to be evaluated within that context. 

The application of these similar deep learning methods across multiple problem domains can be attributed to their ability to learn underlying representations. In an abstract sense, disregarding fine-details of the underlying architecture, a naive and untrained deep learning model only becomes domain specific when fit to a set of data. However, this flexibility and reliance on data can render a model sensitive to unintended signals—as seen in adversarial attacks [26].

## 5. Deep Learning Methods

### 5.1. Sequence to Structure

#### 5.1.1. Antibody

Efforts to improve antibody modeling have primarily focused on determining the structure of the CDRs from their sequence alone. Modeling algorithms, such as homology modeling, have been largely successful at determining the structure of non-H3 CDRs, which mostly fall into canonical structural clusters, determined by length and amino acid sequences for key residues. Machine learning methods such as Gradient Boosting Machines (GBM) and Position Specific Scoring Matrices (PSSM), have been used to learn how to group and classify non-H3 CDRs into structural clusters [27,28]. The strong structural similarity across sequences within the same canonical cluster renders modeling of these sequences relatively trivial. Training of these models is done using curated sets of high-resolution antibody Protein Data Bank (PDB) structures.

The lack of effective modeling approaches and the relative significance of the H3 CDR has led to a number of deep learning algorithms attempting to structurally model the H3 loop. One of these approaches is DeepH3, developed by Ruffolo et al. [29]. Employing deep residual neural networks, DeepH3 is able to predict inter-residue distances (*d*, using C_β_ atoms and C_α_ for glycines) and inter-residue orientation angles (θ, ω as dihedral angles and φ as a planar angle) by generating probability distributions between pairs of residues. The purpose of the model is to look at hypothetical structures of an H3 loop generated by a modeling algorithm, and rank the structures to identify the most likely conformation of the H3. The benchmark dataset came from the PyIgClassify database (with some curation, including removal of redundant sequences) and included only H3 s from humans and mice [30]. For training, 1462 structures were taken from the Structural Antibody Database (SAbDab), with 5% of loops randomly chosen and set aside for validation, to account for overfitting [31].

DeepH3 reports that the Pearson correlation coefficients (r), which simply measures the linear correlation between two variables (in this case the correlation between predicted and target angles) for d and φ were 0.87 and 0.79, respectively, and the circular correlation coefficients (rc) (a circular analogue of the Pearson correlation coefficient) for dihedrals ω and θ were 0.52 and 0.88, respectively. DeepH3 was compared to Rosetta Energy and found an average 0.48 Å improvement for the 49 benchmark dataset structures. Furthermore, they were able to show DeepH3′s discrimination score (D, the model’s ability to distinguish between good and bad structures) superiority over RosettaEnergy with −21.10 and −2.51, respectively. For a case study involving two antibodies and 2800 of their decoy structures, DeepH3 performed significantly better for one (D = −28.68, RosettaEnergy D = 3.39) yet performed slightly worse on the second (D = 0.66 for DeepH3 and D = −1.59 for RosettaEnergy).

#### 5.1.2. Protein

##### AlphaFold

As one might expect, the majority of deep learning approaches for modeling biomolecules have been focused not just on antibodies, but on proteins more generally—primarily in the field of protein fold prediction, which seeks to generate structures from proteins’ amino acid sequences. One such method is AlphaFold, where a protein-specific potential is created by using structures from the PDB to train a neural network to predict the distances between residues’ C_β_ atoms [32]. After an initial prediction, the potential is minimized using a gradient-descent algorithm to achieve the most accurate predictions. AlphaFold uses a dataset of structures extracted from the PDB, filtered using CATH (Class Architecture Topology Homology Superfamily database) 35% sequence similarity cluster representatives, yielding 29,427 training and 1820 test structures. When benchmarked against the Critical Assessment of Protein Structure Prediction (CASP13) dataset, AlphaFold performed best out of all groups, generating high accuracy structures for 24 out of the 43 “free modeling domains”, or domains where no homologous structure is available.

One drawback of the AlphaFold method is the requirement of a multiple sequence alignment which may vary in usefulness across proteins. 

##### Recurrent Geometric Network

A deep learning method which requires only an amino acid sequence and directly outputs the 3D structure was presented by AlQuraishi [33]. In this work, a recurrent neural network is utilized to predict the three torsion angles of the protein backbone. AlQuraishi breaks his method, a recurrent geometric network (RGN), into three steps. In the first step, the computational units of the RGN transform the input sequence into three numbers representing the dihedral, or torsion, angles of each residue along with information about residues encoded in adjacent computational units. This computation is done once forward and then once backward across the sequence, allowing for the model to create an implicit representation of the entire protein structure. 

The three computed torsion angles are then used in the second step to construct the entire structure one residue at the time. In the final stage, the generated structures are scrutinized by comparing them to the native structure. The score used is a distance-based root-mean squared deviation (dRMSD), which allows for utilization of backpropagation in order to optimize the model.

All available sequence and structure data prior to the CASP11 competition was used for training (with a small subset reserved for validation and optimization) and structures used during the actual competition were used for testing the RGN. Results from free modeling (FM, novel proteins) and template-based modeling (TBM, structures with known homologs in the PDB) structures were reported and compared to results of all server (automated) groups from the CASP11 assessment. The RGN outperformed all groups when comparing dRMSD values and was jointly the best when looking at the TM-score in the FM category. For TBM, it does not beat any of the top five groups but lands in the top 25% quantile for dRMSD. These results can be explained by the following advantages and disadvantages: the model is optimized using dRMSD, never sees TM-score during training, and is not allowed to use template-based modeling like the other groups.

Interesting to note is the propagation of solved torsion angles across the sequence from the upstream and downstream calculations of the recurrent neural network. Since the structures of antibody framework regions and non-H3 CDR loops can be modelled relatively easily due to their common canonical structures, the solved torsion angles for the residues which make up these regions could easily be propagated across the residues of the H3 loop during the first stage of the aforementioned method. The minor changes required to implement these modifications make this an attractive framework for H3 modeling.

##### Transform-restrained Rosetta (trRosetta)

Another method for predicting inter-residue orientations and distances uses a deep residual convolutional neural network. Transform-restrained Rosetta (trRosetta) uses the input sequence and a multiple sequence alignment in order to output predicted structural features, which are given to a Rosetta building protocol to come up with a final structure [34]. The network learns probability distributions from a PDB dataset, and extends this learning to orientation features (dihedral angles between residues). After high-resolution checks, a 30% sequence identity cut-off, and other requirements—such as sequence length and sequence homology—a total of 15,051 protein chains were collected and used for training. The network was tested using 31 free modeling targets from CASP13 and compared to the top groups from the modeling assessment. TrRosetta had an average TM-score of 0.625, beating the top server group (0.491) and the top human group (0.587). Further validation was done using 131 “hard” and 66 “very hard” targets from the Continuous Automated Model EvaluatiOn (CAMEO). For the “hard” set, the reported TM-score (0.621) was 8.9% higher than Rosetta and 24.7% higher than HHpredB, the top two groups. The “very hard” set was taken from the 131 targets that had scored less than 0.5 by HHpredB. These structures received an average TM-score of 0.534, 22% higher than Rosetta and 63.8% higher than HHpredB. The trRosetta group notes that, unlike the other teams from the challenge, trRosetta’s tests were not performed blindly and they plan to confirm these improvements in a future protein assessment challenge. Finally, the group looked at the network’s performance on 18 de novo designed proteins and found that their method is considerably more accurate at predicting designed protein structures than structures of natural proteins.

### 5.2. Interaction Prediction/Affinity Maturation

#### 5.2.1. Deep Learning Used for Antibody Lead Optimization 

A successful application of deep learning within the domain of interaction prediction comes from a sequence-based approach proposed to optimize an existing antibody candidate by Mason et al. [35]. Rather than using a public domain dataset, the authors generate a relatively small number of variants (5 × 10^4^) of an existing therapeutic antibody by introducing mutations to the H3 regions of the CDR and screening the variants for binding against a target antigen. The H3 sequences, labeled as binding or non-binding, were used as input to long-term-short-term recurrent neural networks and convolutional neural networks which were trained to predict the binding label of the sequences. Trained networks were then used to filter a computationally generated set of 7.2 × 10^7^ candidate sequences to 3.1 × 10^6^ predicted binders. Experimental testing showed that 30 out of 30 randomly selected predicted binding sequences bound specifically to the target antigen, with one of the thirty exhibiting a three-fold increase in affinity.

The significance of this method is highlighted by the comparative analysis with a structure-based approach. The authors demonstrate that the number of new binding sequence suggestions generated by the structural modeling software is orders of magnitude smaller than the actual expected binding sequence space and that the free energy estimation of the modelled structures could not be used as a reliable classifier of binding activity. 

While structure-based methods have the potential to represent richer features for a given input, sequence-based methods benefit from more data availability due to developed experimental methods such as next-generation sequencing.

#### 5.2.2. Ens-Grad

Another sequence optimization algorithm, Ens-Grad, uses an ensemble of neural networks and gradient ascent to optimize an H3 seed sequence [36]. Briefly, Liu et al. report training an ensemble of six neural networks (five convolutional) using experimental phage display data generated from panning experiments. Panning experiments approximate enrichment in binding affinity by subjecting a set of H3 sequences bound to phages to a binding competition where non-binders are washed away and binders are kept for subsequent rounds [37]. Several different models with varying architectures were trained using either a means squared error loss for regression of enrichment, or a binary cross entropy for classification of H3 CDRs that were successively enriched in rounds of panning. 

After fitting the ensemble of neural networks, the authors use gradient ascent to optimize input seed sequences. Contrary to gradient descent, which is generally used to modify neural network weights so as to minimize a loss function such as classification error, gradient ascent is used in this case to modify the input sequence so as to maximize the output. The authors suggest that the use of an ensemble of several neural networks allows for optimization to take controlled paths by optimizing with respect to different network outputs. 

Using this optimization protocol, the authors were able to generate sequences with greater enrichment than both seed sequences and sequences within the training dataset. This significant result suggests that the neural network models were able to extrapolate beyond input training data, possibly by learning high level representations of what determines enriched binding. 

Additionally, the authors demonstrate superior performance using a gradient ascent method compared to more common generative models such as variational auto-encoders and genetic algorithms. However, it is unclear whether or not the difference between these methods is attributable to the style of optimization, or to the difference in the architecture of the network (e.g., number of layers or layer sizes).

Similar to the method developed by Mason et al. [35], this method completely circumvents the need for structural data which is significantly more difficult to acquire. However, it is highly unlikely that these methods generalize well across target antigens. In each method the network is fit to data points derived from a single target antigen and therefore applying this method to a different target would require extensive wet-lab testing to generate the training data and refit the model.

#### 5.2.3. DeepInterface

DeepInterface is a structure-based method which aims to classify protein complexes in their docked conformational state as either true or false binders [38]. The input to the network is a voxel grid constructed from a fixed-size box placed around the interface. To handle rotation ambiguity, the authors align the vector between the structures center of mass to one of the three coordinate axes. The network itself is composed of four convolutional layers followed by batch normalization and rectified linear units. To transform the voxel space into a one-dimensional vector and subsequently into a prediction of binding, global average pooling is applied to the voxel space followed by two fully connected layers. 

Of note here is the generation of negative data used to train the network. Negative examples in this case refer to any structures which are not true binders. Using negative examples is a vital step in classification, as the network must be exposed to some form of negative input for successful training. To generate these negative examples, the authors use a fast-Fourier transform (FFT)-based docking algorithm, ZDOCK, to select incorrect docking solutions from the set of sampled conformations [13]. 

Representation of the protein interface as a voxel grid is an intuitive yet problematic strategy. Firstly, the input size of the network restricts the voxel space to a single size. The authors overcome this by limiting the size of the interfaces passed into the network to those small enough to fit into the bounded grid space. Secondly, a rotational ambiguity problem arises due to the absence of a common axis across all interfaces. Similar voxel methods used for 3D objects can usually take advantage of an implied gravity vector to eliminate ambiguity across an axis. Handling ambiguity between the remaining axes can be done using a randomly rotated version of the input or by implementing rotational pooling into the network architecture. However, these methods are impractical for more than two dimensions as the number of possible rotations needed grows exponentially. Despite these limitations, DeepInterface achieves 75% classification accuracy on benchmark datasets, demonstrating viability in this classification task 

Due to the differences previously mentioned between antibody-antigen interfaces and general PPIs, it is not clear that the model here presented would be capable of avoiding false positive classification. This problem may give rise to out-of-distribution errors, which arise when the underlying training dataset is not representative of its real-world use case. However, except for the bounding voxel space size, the model architecture and input structure presented within DeepInterface is somewhat agnostic to the type of interface evaluated. It should be noted, however, that the model’s reliance on spatial arrangement of the interface area should not hinder its applicability towards antibody–antigen interfaces, which possess non-discernable differences in shape complementarity [6].

#### 5.2.4. MaSIF-Search

The MaSIF approach comes out of the growing field of geometric deep learning. Starting from a mesh representation of a protein surface, a patch is created by selecting a point on the mesh and all neighboring surface points within a defined geodesic distance [39]. Each of the surface points is annotated with geometric and chemical features which describe degrees of curvature, concavity, electrostatic potential, hydrophobicity and hydrogen bond potential. The patch is down-sampled into a grid of 80 bins (5 radial × 16 angular). Each bin contains the statistical mean of the feature attributes of the points which are assigned to the corresponding bin. The 80 bins, indexed by polar and angular coordinates, are passed as input into a set of geodesic convolutional filters to generate a one-dimensional descriptor of the protein surface. Rotational max-pooling is used to overcome angular ambiguity. The one-dimensional descriptor is then refined by a fully connected layer. The remaining architecture is regarded as application-specific, which expresses the ability to use the 1D descriptors as input into an application specific model.

To train an application specific model for interaction prediction, a modified version of the triplet loss function is used, which minimizes the Euclidean distance between the 1D descriptors of an anchor (a binding protein patch) and a positive (a complimentary patch to the anchor), and maximizes the distance between the anchor and a negative (a randomly chosen, non-complementary surface patch to the anchor). The authors deem two surface patches from two separate proteins to be positive pairs if the patch centers are within a small distance from one another at the protein–protein interface.

To measure the model’s performance, the authors classify interacting vs. non-interacting pairs and report an area under the curve of the receiver operating characteristic (ROC AUC) of 0.99 when using geometric and chemical features. The authors further evaluate the model’s performance on different subsets of the data by creating subsets of low, high and very high interface complementarity. It is interesting to note that, as expected, the classification performance of the model drops to 0.81 ROC AUC using both geometric and chemical feature sets on the low shape complementary subset and further to 0.73 and 0.75 when using only the geometric and chemical features on this subset, respectively.

MaSIF search is trained on a mix of antibody–antigen and protein–protein interfaces with no distinction between the two. As mentioned previously, antibody–antigen interactions exhibit a similar shape complementarity to that of other protein–protein interfaces [6]. This observation provides evidence to suggest similar expectations as those arrived in the investigation of DeepInterface, which is that models capable of capturing geometric matching across protein–protein interfaces should extrapolate well to antibody–antigen interfaces in this regard.

Other machine learning methods, not strictly considered to be deep learning methods, further reinforce this point. As an example, a graph-based machine learning approach called mutation Cutoff Scanning Matrix (mCSM) which predicts changes in affinity upon mutation was developed and evaluated separately on protein–protein and antibody–antigen mutations [40]. The model fit to a protein–protein mutation dataset, mCSM-PPI, performs significantly worse (Pearson coefficient of 0.35) than the model specialized for antibody–antigen interactions (Pearson coefficient of 0.53).

#### 5.2.5. TopNetTree

The need to treat antibody–antigen interfaces as special cases of protein–protein interfaces is further reinforced by the analysis of a deep learning method termed TopNetTree [41]. 

TopNetTree is a recent, innovative approach that uses techniques from persistent homology as a means to represent protein structures as a set of one-dimensional features. Specifically, the use of element-specific persistent homology allows the topological features to be specific to chemical and compositional properties, as well as to atoms within (or a certain distance away) from the mutation site. Using these methods, one-dimensional barcodes are extracted which represent pairwise atomic interactions, the existence of cavities and other multi-atom structures such as loops. Along with the topological features, several other features are included, including solvent accessible surface area, partial charge, and electrostatic solvation free energy.

Mutations are encoded by concatenating the features generated from the native and mutated structure. The first level barcodes, which represent the pairwise atomic interactions, are used as input to a convolutional neural network with four convolutional layers and one dropout layer. The network is trained to minimize the mean-squared error between the final output and ΔΔG. After initial fitting, the output logits of the final convolutional layer are fed into a set of gradient-boosted trees to rank the importance of the convolutional features. The most important features are combined with the higher level topological features as input to a final set of gradient-boosted trees to obtain a final prediction for ΔΔG.

When trained on a subset of the SKEMPI2 database excluding antibody–antigen complexes, and tested on a set of 787 mutations within antibody–antigen interfaces, TopNetTree achieves an R_p_ of 0.53 and a root mean squared error (RMSE) of 1.45 kcal mol^−1^ [42]. When performing 10-fold cross validation on the aforementioned training set, which contains only protein–protein interfaces, the authors report an R_p_ 0.82 and an RMSE of 1.11 kcal mol^−1^. When compared with other predictors of change in affinity upon mutation, TopNetTree exhibits state-of-the-art results for both general protein–protein interfaces as well antibody–antigen interfaces. The performance difference seen between the predictability of ΔΔG in protein–protein and antibody–antigen mutations highlights the need to treat antibody–antigen interfaces as separate and special conditions.

### 5.3. Target Identification

#### 5.3.1. Antibody Specific B-Cell Epitope Predictions

Similar to interaction prediction methods, target identification methods can be separated into two primary classes based upon the input used: structural or sequential. The first of these that we review here is a structural method which demonstrates the increased challenge of predicting interacting domains on an antigen surface without information about the interacting antibody paratope [43]. In this work by Jespersen et al., to formulate a one-dimensional input vector which can be fed into a fully-connected neural network layer, the authors start by defining a patch as a residue and all of its surface-exposed neighbors within a 6 Å proximity. To represent the geometric properties of the patch, the authors use the first three principle components of the C_α_ atoms and Zernike moments. Zernike moments are a particularly noteworthy feature in this work as they function similar to filters of a convolutional neural network by deconvoluting the underlying patch into scalar values representing degrees of particular shapes and patterns found within the patch. Along with these geometric features, compositional features such as solvent exposure and amino acid composition statistics are included.

For training data, the authors construct a negative patch by randomly selecting a non-epitope residue and generating a patch through a Monte Carlo method which iteratively adds neighboring residues to, and removes neighboring residues from, the patch. Target values for patches fall between 0 and 1 and are determined by the amount of residue overlap with a known true epitope. Similarly, negative paratope–epitope patch pairs are generated by matching epitopes to paratopes from different antibody–antigen clusters.

Three models are used: a full model, a minimal model, and an antigen model—each possessing two hidden layers and a sigmoid activation function, and differing only by the size of the input layer. The full and minimal models use patch features from both epitope and paratope and are trained to score pairings while the antigen model uses only epitope features and is trained to score only epitopes. The minimal model, in contrast to the full model, excludes the Zernike moments’ complex structural features.

To compare the three models, the authors construct a test set of 300 negative samples using the aforementioned protocols for each true epitope or epitope/paratope pair across eight different antibody/antigen clusters. Model scores are used to rank the 301 clusters and a F_rank_ score is determined as the percentage of negative samples ranked higher than the positive. Reported scores are 7.4%, 10.9% and 15% for the full, minimal and antigen models, respectively. These results clearly demonstrate the difference in feasibility between predicting epitopes with and without information of a candidate antibody. Although the exclusion of the Zernike moments cannot be directly attributed to the decrease in performance between the full and minimal set (due to inclusion of other features in the difference set), the results do provide evidence that deconvoluting surface patches into a composition of simpler patterns—as is often seen in convolutional neural networks—may be a powerful tool when working with structural data.

#### 5.3.2. MaSIF-Site

As discussed previously, one such method does in fact take the aforementioned approach of surface deconvolution. Namely, the MaSIF approach, which aims to generate one-dimensional fingerprints from surface patches using geodesic convolutional layers [39]. An overview of the MaSIF method is given above in the Interaction Prediction Section 5.2.4. As previously mentioned, these fingerprints can be fed into application specific layers. MaSIF-site is one such application. In contrast to MaSIF-search, the authors report experiments with different network depths by stacking layers of either two or three geodesic convolutional filters. 

Moreover, in contrast to MaSIF-search, the authors do not provide experimental results of performance under geometric and chemical subsets. However, the reported ROC AUC of the model’s classification performance for predicting interacting vs. non-interacting patches is 0.87 ROC AUC per protein. The authors also present more granular results from evaluating the model’s classification performance on proteins with large hydrophobic patches versus proteins with those with smaller hydrophobic patches. The reported performance is 0.87 for large hydrophobic and 0.81 for smaller hydrophobic patches. This is significant in the context of epitopes, as antibody–antigen interfaces tend to have fewer hydrophobic interactions than general protein–protein interfaces. However, the model showed satisfactory results in distinguishing a wild-type, non-antigenic protein patch from a mutated version which has a known antibody binder, suggesting its applicability for identifying epitopes.

#### 5.3.3. Linear B-Cell Epitopes

While it is estimated that approximately 90% of B-cell epitopes are conformational, a significant amount of attention has been placed on predicting linear B-cell epitopes [20]. The first neural network model used for predicting linear B-cell epitopes was established by Saha et al. [44]. Using a relatively standard recurrent neural network architecture which takes as input an amino acid sequence, Saha et al. report prediction accuracies of 65.93% in classifying linear epitope residues from randomly selected and, presumably, non-epitope residues despite a relatively small training set of 700 sequences.

Another straight-forward architecture for linear B-cell prediction uses a fixed size 20 length sequence as input to a fully-connected architecture with two hidden layers and a final softmax output which ultimately transforms the input sequence to a probability score between 0 and 1. As is the case in other applications, it is difficult to compare this model directly with those previously mentioned due to the use of differing datasets. The reported classification accuracy of 68.33% does, however, suggest improvements.

A better comparison of these methods with one another and other non-deep learning epitope predictors was carried out along with the introduction of an additional deep learning model termed a deep ridge regressed epitope predictor (DRREP) developed by Sher et al. (Table 1) [45]. Briefly, the initial layer of the model uses a randomized set of k-mers which are slid across the input sequence and used to compute a matching score with each k-mer and subsequences of the entire input sequence. The inclusion of a second pooling layer renders this procedure similar to a convolution step where the filters are preset to the randomized k-mers. Contrary to what is most commonly implemented in neural network training, the weights of the third layer (a fully connected layer) are computed analytically using ridge-regression. Finally, an output layer is used to provide residue-level predictions of each residue in the sequence. 

Five different datasets were used to benchmark the aforementioned methods. In every case, DRREP showcases the best performance amongst all deep-learning methods and best performance amongst all other predictors except for the Support Vector Matrix (SVM)-based LBTope on the AntiJen dataset

## 6. Datasets/Benchmarks

### 6.1. AB-Bind

The AB-bind dataset is a collection of 1101 mutations for 32 different antibody–antigen structures and includes experimentally-determined changes in binding free energy associated with each mutant, as well as the experimental conditions under which each was tested [46]. While the structure for each native protein complex is provided, it is up to the user of this database to model any changes the mutation causes to the native structure conformation.

### 6.2. AntigenDB

AntigenDB is a database of validated antigens containing structural, sequence, and binding data [47]. In contrast to other antigen databases, AntigenDB contains data on validated antigens—even in the absence of the underlying epitope.

### 6.3. AntiJen

Another antigen data resource is the AntiJen database which is a curated dataset of B-cell and T-cell antigens with experimental annotations, links to published experimental articles, and PDB entries [48].

### 6.4. CAMEO

The Continuous Automated Model EvaluatiOn (CAMEO) platform complements CASP by conducting fully-automated blind prediction assessments each week [49]. The challenge is completed using pre-released sequences of structures, which are to be published to the PDB on their next release. Totaling an average of 100 targets per five weeks, developers are able to more frequently benchmark and validate their methods, which allows other groups access to more benchmarking result data.

### 6.5. CAPRI

The Critical Assessment of PRedicted Interactions (CAPRI) is an annual competition in which participants are invited to submit structural modeling predictions of protein interactions for unpublished crystal structures [50]. Teams are then ranked based on their accuracy. Many papers working in structural modeling prediction frequently test their algorithm by comparing to the top competitors’ performance in the most recent CAPRI competition.

### 6.6. CASP

The Critical Assessment of Techniques for Structure Prediction (CASP) is a biennial competition in which participants are invited to submit structural modeling predictions (from sequence alone) for unpublished crystal structures [51]. Teams are then ranked based on their accuracy. Many papers working in structural modeling prediction (sequence to structure) frequently test their algorithm by comparing to the top competitors’ performance in the most recent CASP competition.

### 6.7. DOCKGROUND

DOCKGROUND is a benchmark dataset of protein–protein complexes, along with generated decoys from the docking algorithm GRAMM-X [52]. The dataset consists of 61 true complexes along with 100 generated decoys (negative).

### 6.8. The Immune Epitope Database (IEDB) 3.0

The IEDB contains information on sequences, experimental data and in some cases structure data for 973,072 linear and discontinuous epitopes [53]. A current filtered search of the IEDB provides 4416 discontinuous epitopes from 826 antigens. Of these, 1318 of the epitopes have a solved 3D structure. The number of linear epitopes is much more numerous, totalling 968,656. However, of these only 961 have a solved 3D structure.

### 6.9. Integrated Protein–Protein Interaction Benchmark 

Contains protein complexes for which bound and unbound structures are solved, which have an X-Ray structure with resolution <3.5 Å, and which are non redundant [54]. A breakdown of the data is as follows: 230 complexes in total (40 antibody/antigen), of which 179 include affinity measurements (33 antibody/antigen). The dataset is further divided into three categories based upon affinity, stressing that lower affinity complexes present more challenging data samples.

### 6.10. PDB 

The Protein Data Bank is perhaps the largest database of structurally resolved biological molecules [55]. It is contributed to by researchers across the globe and funded by the National Science Founcation (NSF), National Institutes of Health (NIH) and Department of Energy (DOE). The database is the source of many curated datasets. 

### 6.11. PDBbind

Updated annually, the PDBbind database currently contains solved structures for 2594 protein–protein complexes extracted from the PDB, along with binding affinity data for each complex [56].

### 6.12. PIFACE

The PIFACE dataset is a set of unique protein-protein complexes [57]. The clusters in the dataset were clustered using structural similarity and graph theory-based clustering methods to identify 22,604 unique interfaces.

### 6.13. PPI4DOCK

PPI4DOCK is another protein–protein complex dataset with decoys generated from the docking algorithm ZDOCK [58]. Structures in this dataset come from homology-modeled structures in an unbound state, along with an experimentally-determined structure for each complex. The decoys in the dataset are labelled using CAPRI standards as either “Incorrect”, “Acceptable”, “Medium” or “High” quality. 

### 6.14. SAbDab

SAbDab is a structural antibody database that is automatically updated on a weekly basis and includes annotations such as affinity data, and other antibody specific annotations such as CDR classifications [31].

### 6.15. SKEMPI

The SKEMPI database is a curation of mutations of proteins which have been structurally resolved and includes changes in binding free energy. As of this writing, the database includes 7085 entries [42].

## 7. Discussion

As mentioned throughout this paper, an emphasis should be placed on treating antibody–antigen interactions as special cases of protein–protein interactions. Some studies reinforce this point directly by providing comparison of their models between general protein and antibody-specific dataset. Others reinforce this point indirectly, such as with the MaSIF method, which shows that protein interfaces which possess smaller hydrophobic regions (characteristic of antibody–antigen interfaces) are more difficult to classify [40,56]. However, working with antibodies does have some advantages. Five out of the six CDR loops have canonical structures, providing a reliable means to infer the structure from the loop sequence, and narrowing modeling efforts to the H3 region. Furthermore, structural properties and makeup make it possible to define a common axis across antibodies, potentially resolving rotation ambiguity problems which arise in structural representations [59].

Though there exist several commonly used datasets for each problem domain, not all methods utilize a single common set. Furthermore, several methods use a refined form of a given dataset or different protocols for clustering and splitting of training, validation and testing data. These differences make it difficult to arrive at exact comparisons between methods. We hope that the curation of the databases mentioned in this paper will bring the field closer to the status of that in other fields such as that of image recognition. As the amount of data and methods continues to grow, initiatives to maintain benchmarks—such as CASP and CAPRI—will become increasingly valuable.

Deep learning requires a significant amount of data. This is demonstrated by its superior performance in domains where large amounts of data are available. While there is indeed a large volume of protein sequence data currently available, structural data still lags behind—due to both the expense of solving protein structure experimentally, and the difficulty of extracting structure from sequence. As antibody–antigen structures are a subset of protein complexes, the amount of antibody structure data is even more limited. Though not applied in any of the aforementioned methods, one strategy to overcome limited antibody–antigen data would be to utilize transfer learning. Transfer learning is the process of retaining knowledge gained during learning from one problem area to another [60]. For example, since protein–protein interactions are governed by similar biochemical principles as antibody–antigen interactions, yet exhibit differing underlying interaction distributions, networks can be initially fit to protein–protein datasets and later refined by a dataset of exclusively antibody–antigen data. This method allows the network to capture the high level principles which govern both phenomena, while leaving room for refinement of the model for antibody-specific applications. A previous example of this technique is the use of a protein folding network trained on general proteins before being applied to the problem of membrane proteins [61].

Due to their evolution, many proteins share structure and sequence similarities. Tools and algorithms—such as BLAST or HMMER for sequence similarity and FATCAT for structure similarity—provide a means to score (and therefore cluster) proteins based on similarity metrics [62,63,64]. While some datasets provide clustered sets, other datasets leave it to the user to generate clusters. Clustering and using representative samples from each cluster can ensure that highly-similar potential cluster members are not distributed across train, validation and test sets. This is an important step in any learning-based methods to mitigate data leakage or inflated results due to overfitting. Protein structural data can be obtained through several methods, but the most common and accurate method is X-ray crystallography. Though it tends to provide better resolution than other methods, it is not perfect. The accuracy of the results of a crystallographic experiment may depend on the type of equipment and solution used as well as the inherent structure and stability of the protein itself. Structures with low resolution are typically less trustworthy in terms of representing the true protein structure, and many have “unsolved” regions where no reliable structural data is available—particularly within highly flexible regions such as the aforementioned H3 loop [65].

Furthermore, affinity measures are sensitive to experimental setup which can differ from experiment to experiment. This can be problematic as this information is often not included in model inputs used to predict these values.

One hurdle that must be overcome is the generation of meaningful negative data for classification problems. For example, with regard to target selection: while protein–protein interfaces are well conserved—and, therefore, non-interacting residues may be reliably labeled as such—antibodies theoretically have the ability to bind virtually any region on a target protein. Because of this, negative labeling is a much trickier problem. In other words, lack of data showing antibodies binding to a certain region is not sufficient evidence that it is impossible for an antibody to bind that region. This means that labeling antigen residues which have not yet been found to interact with any antibody as definitively “non-interacting” could result in a categorical error. Furthermore, even methods which aim to classify interfaces or complexes as binders or non-binders must still be careful about how the negative structures are generated. For example, DeepInterface’s use of docking decoys means that any freedom from out-of-distribution predictions is reliant on the quality of the docking decoys. That is to say, poor docking conformations may be easily separable from truly-binding conformations, and therefore extensive sampling of the virtually infinite negative space is necessary. However, techniques such as those used in MaSIF, where the spatial orientation of two complexes with respect to one another can be wholly ignored, are not subject to the same risk. 

As the field of deep learning continues to stretch into other domains, novel architectures and means of representing input data are likely to follow. These domains will bring with them new tasks which can be reformatted and made relevant to biological applications. For example, the triplet loss function used in the MaSIF method was previously used for the task of face recognition [66]. As for data representation, voxel grids were previously used in object segmentation and recognition [67]. Likewise, recurrent neural networks were used to analyze written text [68]. One strategy to determine whether these novel methods will become applicable to the domain of biologics would be to assess the feasibility of representing the biological data in a format compatible with developing architectures. In this work, we have focused on sequence and structural data. However, biological data is not restricted to these representations. For example, interactions between proteins or similarities between their attributes can be represented as a graph network.

In summary, to adapt these biological data for use with developing architectures, one must first establish a compatible representation which maps into an appropriate format, and critically assess what patterns within the representation may be captured by a deep learning framework.

### 7.1. Future Directions

#### 7.1.1. Structural Representations

Particularly with structure-based methods, the choice of data representation scheme plays an important role in applying a deep learning approach. The growing field of geometric deep learning supplies a number of examples where the representation of structural data in coordinate systems with a local basis both reduces model complexity, and provides invariance to transformations such as rotation. Such methods also circumvent issues that tend to arise in voxel-based techniques, such as data sparsity (most voxels contain empty space) and rigid constraints on the input (such as bounding size of the voxel grid).

#### 7.1.2. Standard Datasets and Benchmarks

Crucial to developing and improving upon new methods in any deep learning field is the availability of a common benchmarking dataset. For example, deep learning applications in small molecule design benefit from commonly used benchmarking datasets such as ZINC and ChEMBL and image-based deep learning methods are often evaluated on MNIST or ImageNet [69,70]. Such datasets are characterized by a large number of data samples so that splits of training, test and validation sets can be made. As the field continues to develop and the volume of additional data grows, it is hoped that there will arise large standard datasets which can be used to directly compare model architectures and techniques and provide a clearer view of the path forward.

#### 7.1.3. Generative Methods 

A deep learning methodology from the field of small molecule discovery and design which has not yet extended to biologics is the application of sophisticated generative frameworks for structure generation. Several methods have been proposed which reliably generate novel stable chemical structures and even allow for the enforcement of particular chemical properties, but these are not applicable to proteins. This difference can be attributed to the larger size and number of complex variations protein structures take on, as compared to small molecules. The development of generative models applicable to biologics could lead the way to networks which are capable of designing paratope structure and composition based directly on a given epitope. 

#### 7.1.4. Deep Learning in Molecular Dynamics

Not touched upon in this review are developments within the intersection of deep learning and molecular dynamics. In short, molecular dynamics simulations aim to reproduce the movement of atoms as they are manifested in reality. Applications of molecular dynamics are broad and revolutions within the field could accelerate discoveries and developments across many protein engineering tasks. For details covering deep learning advances in this area, the reader is directed to a review article on the topic of machine learning and molecular dynamics [71].

## 8. Conclusions

This paper discusses several methods used to improve our understanding of the biochemistry of proteins using the vast trove of biological data which has been created in the past few decades. Deep learning has become an integral part of scientific research in biochemistry at large and is increasingly a component of drug discovery efforts. The growth of biologic drugs—and more specifically, antibodies—as a subject of study makes clear the necessity of adapting deep learning methods to improve outcomes for drug discovery efforts in this field. Antibody drugs have the capability to bind their clinical targets with both high affinity and specificity, and the need for machine learning algorithms specifically adapted to the understanding and enhancement of these proteins is evident.

However, a major challenge remains with regard to the amount and type of protein data available. While there are indeed a large number of protein structures available, the volume of data is completely dwarfed by the proliferation of sequence data made available by modern sequencing technology. The development of new protein structure solving methods may improve this disparity, but for now the comparatively small volume of structural data limits the effectiveness of some deep-learning approaches. Additionally, negative data can be difficult to find—especially for problems like binding prediction, where traditional methods of structure determination can only produce positives. Computationally generating such negative data comes with its own set of challenges; it is difficult to know if generated negatives are indeed true negatives, and it is just as difficult to generate negatives that look sufficiently realistic to begin with. Even within the bounds of well-understood positive data, the disparate nature of the various training datasets used by each of these models makes them difficult to compare and makes it difficult to determine which is optimal for a given application.

As new data and methods arise to confront the current limitations of deep learning and its application to protein science, we will continue to see incremental advancements in our understanding of this challenging space. The translation of these improvements into new understanding in antibody drug development will be vital to the creation and improvement of new medications. The combination of deep learning with antibody science has boundless potential to aid in the creation of new, sophisticated medicines that push the boundaries of current technology.

## Figures and Tables

**Table 1:** Comparison of several linear B-cell epitope predictors across five different datasets. Results taken from [45].

DataSet	Tot Residues	Epitope%	System	75spec	AUC
SARS	193	63.3	**DRREP**	86.0	0.862
			BCPred	80.3	_
			ABCPred	67.9	0.648
			Epitopia	67.2	0.644
			CBTOPE	75.6	0.602
			LBtope	65.8	0.758
			DMN-LBE	59.1	0.561
HIV	2706	37.1	**DRREP**	61.4	0.683
			BepiPred	_	0.60
			ABCPred	61.2	0.55
			CBTOPE	60.4	0.506
			LBtope	61.2	0.627
			DMN-LBE	63.6	0.63
Pellequer	2541	37.6	**DRREP**	62.7	0.629
			LBtope	60.9	0.62
			DMN-LBE	62.8	0.61
AntiJen	66319	1.4	DRREP	73.0	0.702
			**LBtope**	74.2	0.702
			DMN-LBE	_	_
SEQ194	128180	6.6	**DRREP**	75.9	0.732
			Epitopia	_	0.59
			BEST10	_	0.57
			BEST16	_	0.57
			ABCPred	_	0.55
			CBTOPE	_	0.52
			COBEpro	_	0.55
			LBtope	75.3	0.71
			DMN-LBE	_	_
